# Advancements in γδT cell engineering: paving the way for enhanced cancer immunotherapy

**DOI:** 10.3389/fimmu.2024.1360237

**Published:** 2024-03-21

**Authors:** Megan Yuan, Wenjun Wang, Isobel Hawes, Junwen Han, Zhenyu Yao, Alice Bertaina

**Affiliations:** Division of Hematology, Oncology, Stem Cell Transplantation and Regenerative Medicine, Department of Pediatrics, Stanford University, School of Medicine, Stanford, CA, United States

**Keywords:** γδT cells, immunotherapy, engineering, cellular therapy, cancer, CAR-T

## Abstract

Comprising only 1-10% of the circulating T cell population, γδT cells play a pivotal role in cancer immunotherapy due to their unique amalgamation of innate and adaptive immune features. These cells can secrete cytokines, including interferon-γ (IFN-γ) and tumor necrosis factor-α (TNF-α), and can directly eliminate tumor cells through mechanisms like Fas/FasL and antibody-dependent cell-mediated cytotoxicity (ADCC). Unlike conventional αβT cells, γδT cells can target a wide variety of cancer cells independently of major histocompatibility complex (MHC) presentation and function as antigen-presenting cells (APCs). Their ability of recognizing antigens in a non-MHC restricted manner makes them an ideal candidate for allogeneic immunotherapy. Additionally, γδT cells exhibit specific tissue tropism, and rapid responsiveness upon reaching cellular targets, indicating a high level of cellular precision and adaptability. Despite these capabilities, the therapeutic potential of γδT cells has been hindered by some limitations, including their restricted abundance, unsatisfactory expansion, limited persistence, and complex biology and plasticity. To address these issues, gene-engineering strategies like the use of chimeric antigen receptor (CAR) T therapy, T cell receptor (TCR) gene transfer, and the combination with γδT cell engagers are being explored. This review will outline the progress in various engineering strategies, discuss their implications and challenges that lie ahead, and the future directions for engineered γδT cells in both monotherapy and combination immunotherapy.

## 1 Introduction

Immunotherapy has revolutionized cancer treatment, effectively integrating with established medical practices such as surgery and chemotherapy (1, 2). This approach boosts the immune system’s capability to target and eliminate malignant cells, thereby increasing antitumor efficacy and minimizing off-target effects (3). Within the realm of immunotherapy, various strategies have been developed, including the use of immune cells, checkpoint inhibitors, and cytokines. Notably, T cell-based therapies, particularly Chimeric Antigen Receptor (CAR) T cell therapy, have demonstrated significant success against blood cancers (4). In parallel, therapies utilizing NK cells, macrophages, and B cells are emerging as novel treatments for solid tumors and other malignancies (5–7).

Immune cells play crucial roles in the body’s defense mechanisms, including T cells, which are central to cell-mediated immune responses; B cells, which produce antibodies and mediate humoral immunity; and NK cells, which can induce apoptosis in infected or malignant cells as part of the innate immune response (3). Among these immune cells, γδT cells stand out for their unique role in bridging innate and adaptive immunity (8–10). They target and kill cancer cells without the restriction of major histocompatibility complex (MHC) molecules, thus having a broader recognition on cancer cells, including those deficient in MHC class I. γδT cells are adept at secreting cytokines like interferon-γ (IFN-γ) and tumor necrosis factor-α (TNF-α), and they can directly eliminate tumor cells through mechanisms such as Fas/FasL and antibody-dependent cell-mediated cytotoxicity (ADCC) (11). Their ability to migrate to peripheral tissues and respond rapidly to target cells (12), coupled with their lack of involvement in graft-versus-host disease (GvHD) (13, 14), makes them ideal candidates for off-the-shelf cell therapy solutions.

Furthermore, γδT cells are crucial in orchestrating anti-tumor immune responses. They can act as professional antigen-presenting cells (APCs) or influence other APCs like dendritic cells, thereby enhancing the activation of αβT cells and the overall immune response against tumors (15, 16). Theproduction of cytokines, including IL-17 and IL-22, by γδT cells plays a vital role in shaping the tumor microenvironment (TME), thereby influencing tumor growth in various contexts (16–23). This dual role highlights the complexity and importance of γδT cells in tumor immunology and fuels ongoing research into leveraging their therapeutic potential in novel cancer immunotherapies, such as adoptive cell therapy (ACT) (9, 24–27).

Despite their significant therapeutic promise, the clinical application of γδT cells faces challenges. As a minor subset of T cells, they often struggle with *in vivo* survival and proliferation (28), limited persistence, and potential functional suppression upon infiltrating the complex TME (29, 30). To overcome these obstacles, recent advancements in gene-engineering technologies are paving the way for optimizing the therapeutic potential of γδT cells in cancer treatment (8, 9, 15, 28, 31, 32). By genetically modifying these cells to express CARs or enhancing their native T cell receptors (TCRs), their specificity and cytotoxicity against tumor cells can be significantly bolstered. The use of genetic editing tools like CRISPR/Cas9 to knock out inhibitory receptors or to insert cytokine genes further enhances their proliferative and cytotoxic capacities. Concurrently, combination therapies are being explored to enhance the anti-tumor activity of γδT cells, including the use of bispecific antibodies, checkpoint blockade, and cytokine co-administration.

This review aims to deliver a comprehensive overview of cutting-edge approaches to augment γδT cell immunotherapy. It delves into the biological underpinnings and inherent advantages of γδT cells pertinent to their role in immunotherapeutic applications, as well as scrutinizes the forefront of gene-engineering methods being crafted to surmount existing barriers within γδT cell treatment modalities. Additionally, the synergy of gene-modified γδT cells with other treatment modalities is explored, informed by recent clinical research findings. These studies will shed light on the prospective trajectory of γδT cell immunotherapy, underscoring its potential to significantly enhance treatment outcomes for cancer patients.

## 2 Properties and functions of γδT cells

### 2.1 γδT cells ontogeny and γδTCRs diversity

γδT cells are the first T cell lineage to develop in the thymus and can be observed in humans as early as 12.5 weeks of gestational age. However, once generated, these cells will expand and mature extrathymically, and their gene repertoire changes in response to age (33). γδT cells derive their name from their TCRs, which are made up of gamma and delta chains. Like αβT cells, γδT cells undergo somatic V(D)J rearrangement, a process that generates diverse TCRs to respond to a wide range of antigens (34). However, in contrast to αβTCRs, γδTCRs allow cross-reactivity with multiple ligands and each combination is associated with different functional avidities (35). Despite the fact that V(D)J rearrangement of γδT cells generates less diversity than αβT cells, TCR δ chains have a higher potential of diversity at the complementarity-determining region 3 (CDR3) junction and can provide information on a person’s unique history of infection (33, 36, 37).

### 2.2 Tumor targeting mechanisms

γδT cells target real and perceived immunological insults through the production and release of soluble factors. One example of this is when γδT cells recognize pathogen specific antibodies and stress-induced antigens. In response, γδT cells will produce Th1 cytokines including IFN-γ and TNF-α. Subsequently, γδT cells also release cytotoxic granules containing perforin and granzyme, further promoting pathogen degradation (38). Additionally, there is evidence in literature suggesting that Vγ9Vδ2 cells–a subset of γδT cells (discussed in the next section) can act as sensors of a dysregulated isoprenoid metabolism that target specifically cancer cells (39). Moreover, several recent studies have indicated that different subsets of γδT cells may have remarkably different functions in targeting tumor cells (40–43). Therefore, it is important to understand the structure and subsets of γδT cells, which we describe in the next section.

### 2.3 γδT cell subsets

This section will focus on two main subsets of γδT cells: Vδ1 and Vγ9Vδ2.

#### 2.3.1 Vδ1 γδT cells

**Figure 1:**
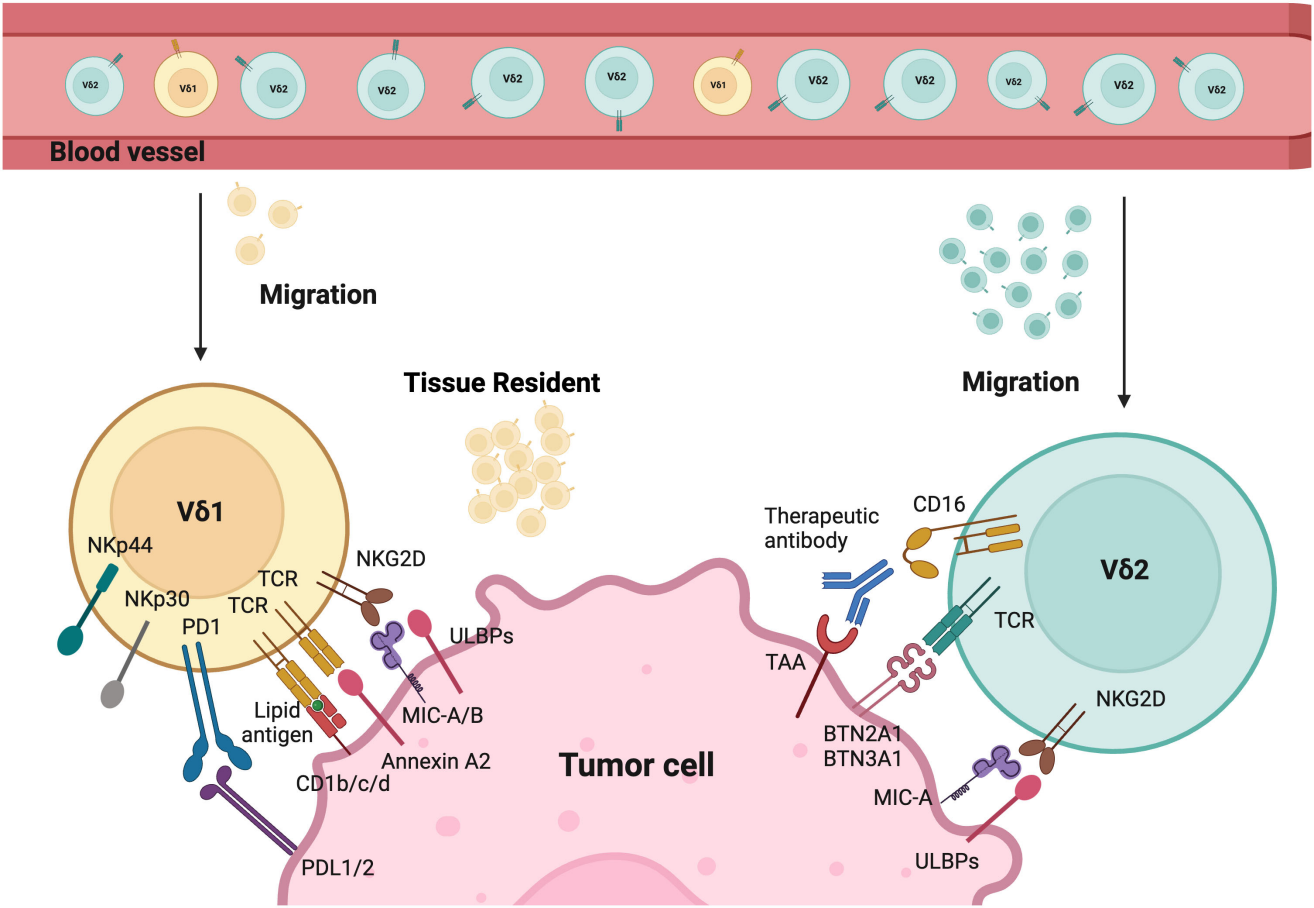
Tumor targeting mechanisms of Vδ1 and Vδ2. Different γδT cells activation modes by tumor cells. The tissue resident Vδ1 T cells recognize cancer cells via their specific Vδ1 T cell receptors (TCRs), which bind Annexin A2 and lipid antigens presented by CD1. Besides, Vδ1 T cells also use NKG2D and natural cytotoxicity receptors (NCRs) such as NKp30, NKp44, and NKp46 for tumor cell recognition. Vδ2 T cells are predominant in the peripheral blood and can migrate into tumor tissues. Their specific Vδ2 TCRs recognize BTN3A1 and BTN2A1 after the isopentenyl pyrophosphate (IPP) accumulation. CD16 expressed by Vδ2 T cells can bind therapeutic antibodies to trigger Vδ2-mediated antibody-dependent cell-mediated cytotoxicity (ADCC). In addition, both Vδ1 and Vδ2 T cells express natural killer receptors (NKRs), which recognize tumor cells by binding to MHC class I chain-related protein A and B (MICA/B), and UL16-binding proteins (ULBPs). Created with BioRender.com.

Vδ1 T cells are primarily localized in various human tissues, particularly abundant in the intestine, skin, spleen, and liver (44) (**Figure 1**). Their properties, particularly their inherent tissue-specific adaptations, have attracted growing interest in the context of cancer immunosurveillance and immunotherapy applications. Phenotypically, these tissue-resident Vδ1 T cells express homing chemokine receptors (e.g., CXCR3, CXCR6) as well as tissue-retention markers (e.g., CD69, CD103, and CD49a) (45–47). Intratumoral Vδ1 T cells have been detected in several solid tumors, exhibiting features of tissue-resident memory T cells (T_RM_) (46, 47). There are also peripheral Vδ1 cells that preferentially express CCR5, CCR6, and CXCR3 (48).

Additionally, Vδ1 T cells have private TCR repertoires and significant TCR diversity that mainly originate from *TRD* repertoires (49). They also manifest features of adaptive immunity, including long-lasting functional memory in γδT cells and adaptive clonal expansion, particularly in response to viral infections (49–51). Studies demonstrate that Vδ1 T cells recognize tumor antigens or cell stress signals through γδTCR and various activating receptors shared with NK cells. These include NK group 2 member D (NKG2D), natural cytotoxicity receptors (NCR, such as NKp30, NKp44, NKp46), and coactivating/adhesion DNAX-activating molecule (DNAM-1) (52–56). Their ligands are frequently expressed on stressed neoplastic cells, for instance, MHC class I chain-related protein A and B (MICA/B), UL16-binding proteins (ULBP) 1-4 are common ligands for NKG2D. Vδ1+ T cells can be directly activated through NKG2D upon the expression of its ligand (e.g., MICA) on tumors, without the need for overt TCR stimulation as seen in αβT cells (55, 57). Moreover, the expression of NCRs on Vδ1 T cells is correlated with increased granzyme B and enhanced cytotoxicity against lymphoid leukemia cells (55). Evidence in the literature suggests that Vδ1 T cells can recognize stress-induced antigens including non-classical MHC class I-like molecules, such as CD1 family (including CD1c, CD1d), MICA/B, ULBP molecules (including ULBP3), and annexin A2 (52–54, 58–62). Interestingly, Vδ1+ T cells are less susceptible to activation-induced cell death (AICD) compared to Vδ2+ T cells. Despite variations in their antigen recognition, both Vδ2 and Vδ1 T cells share similar cytotoxic mechanisms via the perforin/granzyme-B mediated secretory pathway and death receptor pathways such as TRAIL/TRAIL-R, Fas/FasL (38).

Recognition of CD1d is dependent on the presence of lipid and glycolipid on foreign antigens, suggesting that Vδ1 T cells could recognize these antigens in a lipid-dependent manner (63). Bai et al.’s study directly demonstrates this principle of antigen presentation of MHC and lipid recognition by Vδ1 T cells (64). However, the exact mechanism of CD1d recognition in Vδ1 T cells is still unclear and remains an area of continued investigation.

Furthermore, MICA, a stress-induced antigen, triggers activation and expansion of Vδ1 subset via NKG2D when it is expressed on the surface of tumor cells (52–54). These cells have also been shown to recognize ULBP3, a “kill me” signal, expressed on leukemic B cells, suggesting an additional mechanism through which these cells can participate in anti-tumor immune regulation (60).

With advances in innovative isolation techniques and deepening comprehension of Vδ1 T cells, these cells hold high promise as a potential candidate for cancer immunotherapy, particularly as tissue-associated or tumor-infiltrating lymphocytes. Their manipulation using well-designed cell engagers or immune checkpoint inhibitors *in situ* represents an accessible and cost-effective approach. In the future, the use of single cell sequencing/proteomics techniques will be essential to dissect the heterogeneity and functional plasticity of Vδ1 T cells shaped by the TME, thereby aiding their clinical implementation.

#### 2.3.2 Vγ9Vδ2 γδT cells

Vγ9Vδ2 (Vδ2) T cells are among the most studied subsets of γδT cells, partially because these cells represent the most abundant subset in peripheral blood (**Figure 1**). Vγ9Vδ2 cells are generally considered as the first line of defense, forming an essential part of the innate immunity. All Vγ9Vδ2 T cells consist of a public Vγ9 chain and private Vδ2 chain. However, Vδ2 T cells can be further divided into two subclasses (Vγ9+Vδ2+ and Vγ9-Vδ2+) that exhibit distinct properties. Vγ9+Vδ2+ T cells exhibit innate characteristics, while Vγ9-Vδ2+ T cells show adaptive features and undergo pathogen-driven differentiation similar to conventional CD8+ T cells (44, 65, 66).

Similarly, the recognition of Vδ2 cells is mediated by γδTCR or NK cell-activating receptors such as NKG2D and DNAM1. These cells are unique due to their semi-invariant property that allows recognition of specific antigens. Vδ2+ TCRs are capable to recognize phosphoantigens (P-Ag), non-peptide antigens that accumulated in tumor cells due to their dysregulated mevalonate pathway (67). The activation of γδ T cells is intricately linked to the recognition of P-Ag. This process heavily involves the proteins butyrophilin 2A1 (BTN2A1) and butyrophilin 3A1 (BTN3A1). BTN2A1 binds to Vγ9+ γδTCRs. BTN3A1 acts as a critical mediator by presenting P-Ag to γδ T cells through its intracellular B30.2 domain (68). This interaction is pivotal for initiating the downstream signaling pathways that lead to γδ T cell activation and immune responses. Furthermore, Vγ9Vδ2 T cells have distinct patterns of development in fetus and adults. Fetal Vγ9Vδ2T cells are generated in the fetal thymus, while adult Vγ9Vδ2 T cells are developed after birth in response to environmental stimuli and expanded polyclonally by microbial P-Ag exposure (37, 69). The CD16+ Vδ2 T cells can also mediate ADCC upon binding to tumor-specific antibodies, which is absent in Vδ1 T cells (70). Additionally, these cells can function like professional APCs by phagocytosing and processing target antigens, then presenting them with MHC molecules. This process, in turn, induces CD4+ and CD8+ responses in αβT cells (16, 71–73).

Recent studies suggest both subclasses of Vγ9Vδ2 T cells play key roles in the immune defense against pathogens and tumor cells. The number of Vγ9Vδ2 T cells increases dramatically during some infections and these cells display potent cytotoxic activity. During stimulation with non-peptidic antigens, Vγ9Vδ2 T cells can be activated via a dual mechanism involving the recognition of FcγRIIIa (CD16a) following the TCR-CD3 complex, which are cell surface antigens for T lymphocytes and NK cells (74). This activation schema belies a keystone role for Vγ9Vδ2 T cells in the defense of pathological infection as well as tumorigenesis.

## 3 Sources of γδT cells and their expansion strategies

### 3.1 Sources of γδT cells

**Figure 2:**
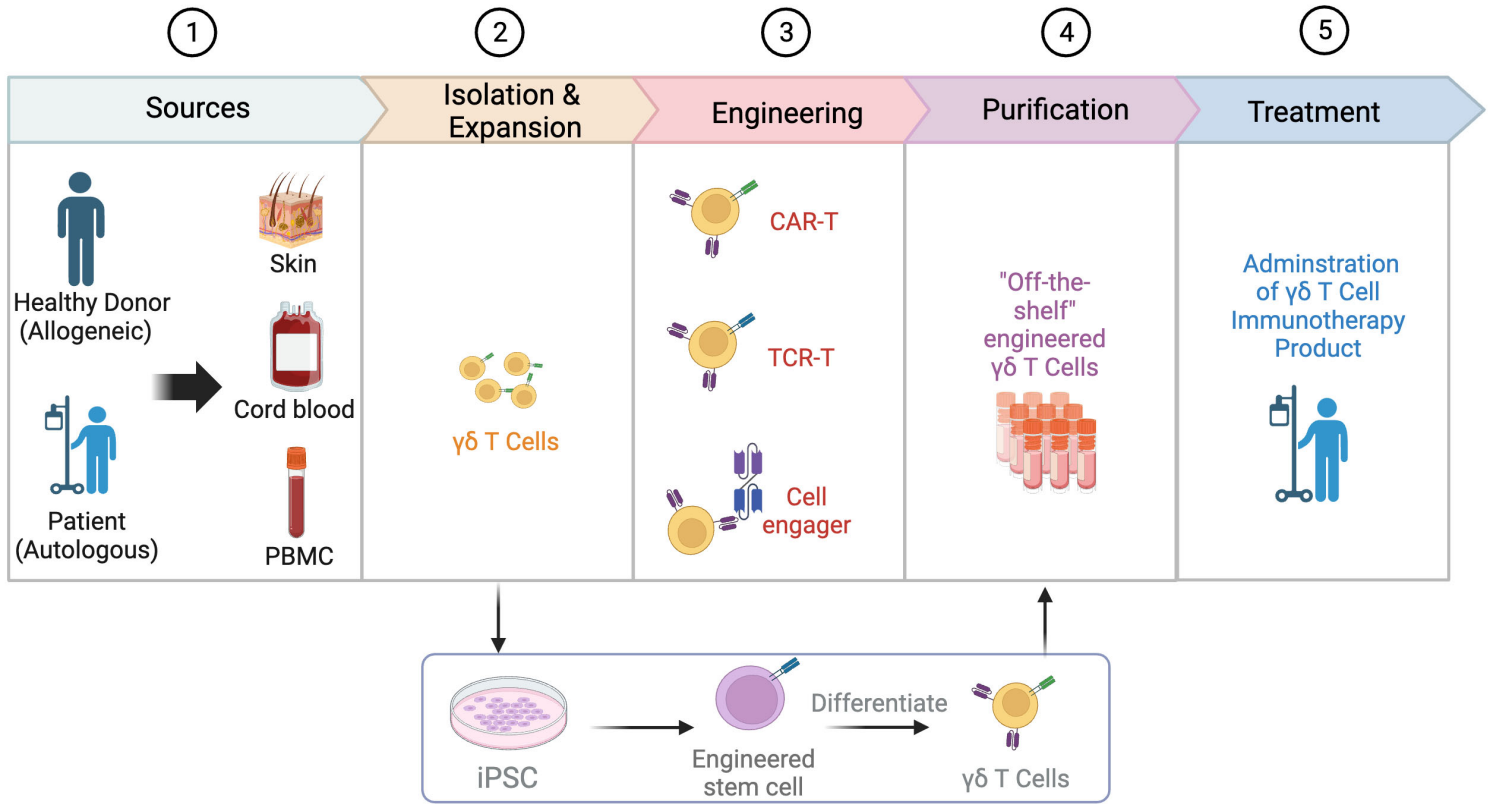
Process of engineering γδT cells. The process of engineering γδT cells involves several key steps. Common sources of γδT cells include the skin, cord blood, and peripheral blood mononuclear cells (PBMCs), with the allogeneic pathway involving isolation from a healthy donor and the autologous pathway involving isolation from the patient’s own cells. After isolation, γδT cells are expanded and engineered through various strategies such as the use of chimeric antigen receptors (CARs), T cell receptor (TCR) transfer, and cell engager. Engineered γδT cells can also be derived from induced pluripotent stem cells (iPSCs). In the next step, γδT cells go through purification to develop “off-the-shelf” engineered γδT cells. Finally, the engineered γδT cell product is administered to patients as a form of immunotherapy. Created with BioRender.com.

The successful clinical application of γδT cell-based immunotherapy must address several challenges, starting with the selection of appropriate sources (**Figure 2**). Inconsistent effects of autologous γδT cells have prompted investigators to design standardized cell products. Because HLA-matching is not required, fully allogeneic mismatched or haplo-identical γδT cells sourced from healthy donors have emerged as an appealing approach with a commendable safety profile (75, 76). A thorough investigation into the donor’s infection history can also benefit patient outcomes when used as a screening criterion. For instance, the reactivation of cytomegalovirus (CMV) in patients receiving HSCT can potentially induce the expansion of Vδ2^neg^ γδT cell clones, which exhibit dual reactivity to CMV and acute myeloid leukemia (AML) (77–79). Another challenge lies in determining which γδT cell subset will be more effective for a specific tumor considering their differing characteristics, particularly their chemotaxis ability and tumor cytotoxicity. Up to now, the main sources of γδT cells include cord blood, peripheral blood, skin, and inducible pluripotent stem cells (iPSCs).

The developmental trajectory of γδT cells reveals that Vδ1+ cells constitute the predominant population (approximately 50%) of γδT cells in cord blood at birth, while Vδ2+ cells typically represent 25% (80). Over time, Vγ9Vδ2 T cells emerge as the predominant subset (over 75%) of the γδT cell population in peripheral blood by adulthood, with less than 10% being Vδ1+ (80). Therefore, cord blood has been explored for its predominant expansion of Vδ1+ cells, or occasional viable expansion of Vδ2+ cells (81–83). However, there are several challenges associated with the *in vitro* expansion of γδT cells from cord blood, including a low number of γδT cells (less than 1% of cord blood lymphocytes), phenotypically and functionally immature γδT cells, and a poor response to IL-2 and phosphoantigen stimulation (80). In contrast, γδT cells isolated from peripheral blood mononuclear cells (PBMCs) are predominantly Vγ9Vδ2 (84). Due to their relative convenience and availability, PBMCs provide easy and stable access for expanding Vγ9Vδ2 T cells and viable Vδ1+ cells such as Delta One T (DOT) cells. Additionally, owing to natural tissue tropism of Vδ1+ cells, human tissues such as skin also provide an alternative source of Vδ1+ cells through enzymatic digestion or other methods (85, 86). Despite the roles of skin γδT cells in the cutaneous malignances such as melanoma, complex skin γδT cell subsets necessitate a thorough investigation for therapeutic strategies (87).

In addition to γδT cells derived from donors, these cells can also be generated from iPSCs (88, 89). Two companies, Century Therapeutics and CytoMed Therapeutics, have developed platforms that enrich γδT cells from healthy donor leukapheresis and then reprogram them into T cell-derived iPSCs (TiPSCs). TiPSCs are engineered with CAR expression, followed by directed differentiation into γδ CAR-T cells (**Figure 2**) (90). During this process, the genome characterization of a single CAR-TiPSC clone enables the production of a highly uniform clonal γδ CAR-T cell bank (> 95% CAR expression) and minimal DNA mutation caused by engineering (90, 91). This off-the-shelf platform provides an appealing source of γδT cells with several benefits: overcoming quantitative limitations of γδT, reducing the wait time for *ex vivo* expansion of γδT cells, and not relying on the *ex vivo* expansion efficiency of PBMC-derived γδT cells (92). Importantly, TiPSC-derived γδT cells retain cytotoxicity to solid and blood tumor through both γδTCR and NKG2D (92). However, this complex manufacturing process is time-consuming and needs more evaluation on potential risks.

Overall, most of the research is adopting PBMC and cord blood as the primary source of γδT cells. In contrast, investigations into skin-derived and iPSC-derived γδT cells are still in the preclinical stages. Our current understanding of the migration and colonization of γδT cells in peripheral tissues primarily relies on research conducted in mice. Further studies involving humans will significantly advance our comprehension of tissue-specific γδT cells, potentially expanding the applications of Vδ1+ cells in immunotherapy.

### 3.2 Strategies to expand γδT cells: prerequisite for therapeutic infusion

The clinical-scale manufacturing of γδT cells requires robust and highly reproducible expansion methods that meet good manufacturing practice (GMP) standards. Current approaches mainly include cytokine only, synthetic p-Ag and bisphosphonate (BP) stimulation, antibody-based expansion, and feeder cell-based strategies as summarized in **Table 1**. Undoubtably, cytokine combinations strategies simplify the manufacturing process but often produce insufficient expansion.

**Table 1:** Comparison of different methods for γδ-T cell expansion.

Source	Expansion strategy	Expand subsets	ref
Tumor specimens	Anti-MICA antibodies	Vδ1	(54)
Healthy donor PBMCs and patient derived	PHA and IL-7	Vδ1	(93)
Healthy donor PBMCs	DOT	Vδ1	(94)
Healthy donor PBMCs	4–1BB	Vδ1	(95)
Healthy donor PBMCs	Mitogen Con A	Vδ1	(96)
Healthy donor PBMCs	anti-CD3 mAb (clone: OKT-3) and IL-15	Vδ1	(97)
Patient-derived	ZOL and BrHPP	Vγ9Vδ2	(98)
Healthy donor and lung cancer patient PBMCs	PTA	Vγ2Vδ2	(99, 100)
PBMCs	IPP and IL-2	Vγ9Vδ2	(101)
Healthy donor PBMCs	Aminobisphosphonates	Vγ9Vδ2	(102)
Healthy donor PBMCs	IL-2 and IL-15	Vγ9Vδ2	(84)
Healthy donor PBMCs	IL-2 or IL-15 combined with TGF-β	Vγ9Vδ2	(103)
Healthy donor PBMCs	Costimulation of ZA and IL-2 in addition to aAPC	Vγ9Vδ2	(104, 105)
Healthy donor PBMCs	Vitamin C with IL-2, ZOL, and HMBPP	Vγ9Vδ2	(106)
Healthy donor PBMCs	CD40L/pp65 and pp65 aAPCs	Polyclonal with predominant Vδ1 phenotype	(107)
Healthy donor PBMCs	K562 feeder cells	Polyclonal γδ	(108, 109)
Healthy donor PBMCs	OKT3	Polyclonal γδ	(110)
Healthy donor PBMCs	anti-TCRγδ antibody	Both	(111, 112)
PBMCs	ZOL	Not specified	(113)
Healthy donor PBMCs	ZOL, IL-2, and IL-18	Not specified	(114)

aAPCs, artificial antigen-presenting cells; BrHPP, bromohydrin pyrophosphate; Con A, concanavalin A; DOT, Delta One T; HMBPP, (E)-4-hydroxy-3-methyl-but-2-enyl pyrophosphate; MICA, MHC class I chain-related protein A; NB, neuroblastoma; OKT3, anti-CD3 antibody; PHA, phytohemagglutinin; PTA, tetrakis-pivaloyloxymethyl 2-(thiazole-2-ylamino) ethylidene-1,1-bisphosphonate; ZOL, zoledronate.

p-Ag or BPs have been recognized as the most established approaches to selectively expand Vδ2+ γδT cells (9). Zoledronic acid (ZOL), a BP, has been widely used to numerically expand Vγ9Vδ2 T cells *in vivo* and *ex vivo*. ZOL can be used alone or in combination with IL-2 to achieve these effects (115). ZOL (5 uM) and IL-2 (1000IU/ml) administration over 14 days has been reported to initiate an over 4000-fold proliferation and expansion of γδT cells (mainly Vγ9Vδ2) from PBMCs of both healthy donors and patients with advanced non-small cell lung cancer (116). However, the expansion folds and purities of γδT cells vary in different published results.

Current protocols for expanding Vδ1+ T cells *in vitro* primarily rely on mitogenic plant lectins such as phytohemagglutinin (PHA) or concanavalin-A (ConA), which induce AICD in Vγ9Vδ2 T cells (93, 117). To transition from the laboratory to the clinic, more efforts have been made to avoid potentially hazardous components. Almeida et al. first developed a clinical-grade two-step method through combination of cytokines (IL-1β, IL-4, IL-21, and IFN-γ) and anti-CD3 mAb (clone: OKT-3) to achieve the expansion of Vδ1+ T cells (94). This method enables large-scale expansion (up to 2,000-fold) of Vδ1+ T cells known as DOT cells (94). GDX012, based on DOT cells, has been granted orphan drug designation by FDA for AML treatment and is currently undergoing evaluation in a phase I trial (NCT05001451). Recently, Ferry et al. also apply only anti-CD3 mAb and IL-15 to stimulate αβTCR- and CD56-depleted PBMC, resulting in robust Vδ1 cell expansion (97).

The feeder cell-based method utilizing artificial antigen-presenting cells (aAPCs) has been explored to provide γδT cells with a sustained activation and costimulation signal. K562, a human chronic erythroleukemic cell line lacking MHC expression, is primarily used as aAPCs. These cells are engineered with costimulatory molecules (like CD80, CD86, CD137) and antigens (e.g., CMV antigen-pp65), allowing for the targeted expansion of specific γδT cell subsets (108, 118). Deniger et al. first activated and propagated polyclonal γδT cells utilizing K562-based aAPCs as irradiated feeders (108, 118). This method requires the additional labor-intensive manufacturing process of culturing feeder cells, yet it mitigates the AICD effects in γδT cells associated with prolonged antigen exposure. Additionally, methods of removing all residual feeder cells before infusion remains a hurdle to clinical implementation of this approach. To address this, several solutions have been proposed, such as gamma-irradiation of aAPCs and the transduction of aAPCs with an inducible suicide gene (107). The *ex vivo* aAPC expanded donor-derived γδT cells are under evaluation of safety and cell dose in a phase I/II trial (NCT05015426) in patients with high-risk acute leukemia (104).

In the future, efforts should focus more on eliminating the use of xenogeneic serum and feeder cells and integrating GMP/pharmaceutical-grade reagents into the expansion process. An example of such a method is the protocol proposed by Bold et al. in a recently published article, which has shown better outcomes in terms of expansion and purity (119). Further efforts can be directed towards enhancing the rate of γδT cell expansion, optimizing the procedure, and lowering manufacturing costs. Besides assessing quantity, evaluating the quality of expanded γδT cells–such as memory and exhaustion phenotypes, is crucial for maximizing therapeutic efficacy and requires further investigations.

## 4 Engineering strategies: the advances and advantages of γδT cell-based immunotherapy

To date, the pharmaceutical industry has explored three primary categories of strategies for γδT cell engineering, which encompass: (1) CAR-T therapy; (2) antibody-based approaches, such as cell engagers or bispecific antibodies; and (3) engineering or transfer of TCRs. CAR-T therapy remains the predominant approach, while antibody-based strategies are gaining prominence due to several advantages. Research is ongoing to investigate combination of therapies aimed at maximizing the unique capabilities of γδT cells. Lists of engineering strategies and ongoing clinical trials are presented in **Tables 2**, **3**, respectively.

**Table 2:** Different strategies for engineering γδT cells.

Product	γδT Source	Subsets	Disease	Transduction methods	Ref
CAR-T					
ADI-002 (Allogeneic GPC3-CAR-γδT Cell)	Healthy donor PBMCs	Vδ1	Solid tumors	γ-retrovirus	Adicet Bio, Inc (120)
ADI-925 (Enhanced intracellular DAP10 chimeric adaptor protein)	Donor PBMCs	Vδ1	Hematologic and solid tumor	–	Adicet Bio, Inc (121)
ADI-270 (CD27-derived CAR-γδT)	Healthy donor PBMCs	Vδ1	CD70+ cancers	–	Adicet Bio, Inc
NKG2DL-targeting CAR Vγ9Vδ2T	Autologous/Allogeneic PBMC	Vγ9Vδ2	Solid tumors	mRNA electroporation	(122)
ns19CAR γδT	Healthy donor PBMCs	Vγ9Vδ2	B cell leukemias	Lentivirus	IN8bio (123)
TMZ and MGMT-modified γδT cells	Healthy donor PBMCs	Vγ9Vδ2	Glioblastoma	Lentivirus	(124)
γδCAR-T cells	Healthy donor PBMCs	Not specified	Leukemia	Retrovirus	(125)
BCMA—Specific CAR	Healthy donor PBMCs	Vγ9Vδ2	MM	mRNA electroporation	(126)
ACTallo^®^	Healthy donor PBMCs	Vγ9Vδ2	N/A	CRISPR gene editing	Immatics
MUC1-Tn-targeting CAR-Vγ9Vδ2T cells	Healthy donor PBMCs	Vγ9Vδ2	Solid tumors	Lentivirus	(127)
Vδ1 T cells engineered with a GPC-3 CAR and sIL-15	Healthy donor PBMCs	Vδ1	HCC	Retrovirus	(128)
CD5-NSCAR- and CD19-NSCAR-γδT cells	Healthy donor PBMCs	Vγ9Vδ2	T-ALL and B-ALL	Lentivirus	(129)
iPSC-derived γδ CAR-T (γδ CAR-iT)	Allogeneic γδT cell-derived iPSCs	Vγ9Vδ2	Hematological and solid tumors	CRISPR gene editing	Century Therapeutics (90)
CNTY-102 (iPSC-derived γδ anti-CD19 and CD22 CAR-T)		Not specified	relapsed, refractory B-cell lymphoma and other B-cell malignancies	CRISPR gene editing	Century Therapeutics
CNTY-107 (iPSC-derived γδ anti-Nectin-4 CAR-T)		Not specified	Solid tumor	CRISPR gene editing	
Anti-GD2 Co-stimulation-Only CAR	Healthy donor PBMCs	Vγ9Vδ2	Neuroblastoma	Retrovirus	(130)
CD123-specific CAR	Healthy donor PBMCs	Vγ9Vδ2	AML	mRNA electroporation	(131)
CD5 -non-signaling CAR (NSCAR), CD19-NSCAR	Healthy donor PBMCs	Vγ9Vδ2	T-ALL and B-ALL	Lentivirus	(129)
T cell engager and bispecific Abs					
CD40-bispecific γδT cell engager	N/A	Vγ9Vδ2	B-cell malignancies	N/A	(132)
CD1d-specific Vγ9Vδ2-T cell engager	N/A	Vγ9Vδ2	CLL	N/A	(133)
Bispecific Antibody Targeting Both the Vγ2 TCR and PD-L1	N/A	Vγ9Vδ2	Solid tumors	N/A	(134), Wuhan YZY Biopharma Co., Ltd
GADLEN (bispecific γδ T cell engagers containing heterodimeric BTN2A1/3A1 extracellular domains)	N/A	Vγ9Vδ2	B-cell lymphoma	N/A	Shattuck
Her2/Vγ9 antibody	N/A	Vγ9Vδ2	Pancreatic cancer	N/A	(135)
Anti-TRGV9/anti-CD123 bispecific antibody	N/A	Vγ9Vδ2	AML	N/A	(136)
EGFR-Vδ2 bispecific T cell engager	N/A	Vγ9Vδ2	EGFR-Expressing Tumors	N/A	(137)
TCRs engineering or transfer					
γδT cells transduced with the αβTCR and CD8 αβ genes	Healthy donor PBMCs	Vγ9Vδ2	MAGE-A4-expressing tumor	Retrovirus	(138)
αβTCRs engineered γδT cells	Healthy donor PBMCs	Not specified	Leukemia	Retrovirus	(139, 140)
TCR transfer combined with genome editing	Healthy donor PBMCs	Vγ9Vδ2	B cell leukemias	CRISPR/Cas9 Lentivirus	(141)
KK-LC-1-specific TCR-transduced γδT cells	Healthy donor PBMCs	Not specified	Lung cancer	Retrovirus	(142)
NKT cell TCR-transfected γδT cells	Healthy donor PBMCs	Vγ9Vδ2	Not specified	Electroporation	(143)

AML, acute myeloid leukemia; CLL, chronic lymphocytic leukemia; EGFR, epidermal growth factor receptor; HCC, hepatocellular carcinoma; MAGE-A4, melanoma antigen-A4; MM, multiple myeloma; T-All and B-All, T and B cell acute lymphoblastic leukemia. N/A, not applicable.

**Table 3:** Summary of ongoing clinical trials of engineered γδT products.

Product	Source & subset	Disease	Clinical Trial ref	Phase	Outcome	Company
CAR-T therapy						
ADI-001 (Anti-CD20 Allogeneic Gamma Delta CAR-T)	Leukapheresis from healthy donor (Vδ1)	B cell malignancies	NCT04735471	I	No GvHD; 3/6 patients had AESIs	Adicet Bio, Inc (10, 144)
ADI-001	Allogeneic	Lymphoma	NCT04911478	N/A	N/A	Adicet Bio, Inc
CD19-CAR-γδT cells	Allogeneic	B Cell Malignancies	NCT02656147	I	N/A	Beijing Doing Biomedical Co., Ltd.
CD19-CAR-γδT cells	Allogeneic	NHL	NCT05554939	I/II	N/A	Chinese PLA General Hospital
Allogeneic NKG2DL-targeting CAR γδT Cells (CTM-N2D)	PBMC from healthy donor	Advanced Solid Tumors or Hematological Malignancies	NCT05302037	I	N/A	CytoMed Therapeutics Pte Ltd
NKG2DL-targeting CAR-grafted γδT Cells	Haploidentical/Allogeneic	Solid Tumor	NCT04107142	I	N/A	
Universal Dual-target NKG2D-NKp44 CAR-T Cells	N/A	Advanced Solid Tumors	NCT05976906	I	N/A	Zhejiang University
CD7-CAR – γδT Cells	Unknown	CD7^+^ T cell-derived malignant tumors	NCT04702841	Early Phase 1	N/A	PersonGen BioTherapeutics (Suzhou) Co., Ltd.
Generation of CD33-CD28 γδT Cells	Vδ2 from peripheral blood and bone marrow	AML	NCT03885076	N/A	N/A	TC Biopharm
Universal CAR-γδT Cell Injection targeting CD123	Allogeneic	AML	NCT05388305	N/A	N/A	Hebei Senlang Biotechnology Inc., Ltd.
Universal CAR-γδT cell	Allogeneic	AML	NCT04796441	N/A	N/A	Hebei Senlang Biotechnology Inc., Ltd.
Cell engager and bispecific antibodies						
LAVA-051 (Vγ9Vδ2-T cell engaging bispecific antibody)	N/A	CLL, MM, AML	NCT04887259	I/IIa	Dose level of 45µg without CRS or DLTs	LAVA Therapeutics (145)
LAVA-1207 (bispecific Vγ9Vδ2-T cell engager)	N/A	Prostate Cancer	NCT05369000	I/IIa	Dose level of 40µg without DLTs; 3/8 patients SD at 8 weeks	LAVA Therapeutics (146)
ET019003 (anti-CD19 Fab - TCR-γδT cells)	N/A	CD19+ Leukemia and Lymphoma	NCT04014894	I	50% (6/12) complete response and 33% (4/12) partial response	Wuhan Union Hospital, China (147)
ACE1831 (allogeneic αCD20-conjugated Vδ2 T cells)	PBMC from healthy donor	Relapsed/​ Refractory CD20-expressing B-cell Malignancies	NCT05653271	I	N/A	Acepodia Biotech, Inc. (148)
ICT01 (anti-BTN3A antibody)	N/A	Advanced solid or hematologic tumors	NCT04243499	N/A	Dose level of 700µg without CRS or DLTs in 6/6 patients	ImCheck Therapeutics
TCRs engineering or transfer						
GDT002 (Vγ9Vδ2TCR-bearing αβT cells)	PBMC from healthy donor	Multiple myeloma	NCT04688853	I/II	N/A	GADETA
Combination therapy						
INB-200 (MGMT modified γδT +TMZ	Autologous	Glioblastoma	NCT04165941	I	No CRS, DLTs, or ICANS in 15/15 patients	In8bio Inc. (149)
INB-400 (MGMT modified γδT +TMZ)	Autologous/allogeneic	Glioblastoma	NCT05664243	Ib/II	N/A	In8bio Inc.

AESIs, adverse events of special interest; AML, acute myeloid leukemia; CLL, chronic lymphocytic leukemia; CRS, cytokine release syndrome; DLBCL, diffuse large B cell lymphoma; DLTs, dose limiting toxicities; ICANS, immune effector cell-associated neurotoxicity syndrome; MM, multiple myeloma; NHL, non-Hodgkin lymphomas. N/A: not applicable.

### 4.1 γδ CAR-T cell therapy: extend from but exceed the conventional αβ CAR-T therapy

CAR-T therapy, with its potential for HLA-independent tumor antigen recognition, has found its place as a key player in cancer immunotherapy. Traditionally, αβT cells have been the main candidates for CAR development (150). However, despite their effectiveness, these cells present several limitations. They are susceptible to GvHD, can cause severe and potentially lethal toxicities, contribute to the development of cytokine release syndrome (CRS), and pose issues related to antigen escape (150). These challenges have spurred an interest in alternative solutions, with γδT cells showing potential to offset these limitations.

Given the wealth of limitations associated with αβT cells, γδT cells are garnering interest as an alternative for CAR-T therapy. These cells do not instigate GvHD, curb antigen escape resulting in decreased relapse rates, and retain beneficial traits such as a less differentiated phenotype with enhanced antigen presentation capacity (125, 151). With these advantages, γδ CAR-T cells may have the potential to overcome the obstacles that have historically troubled conventional αβ CAR-T therapy.

The primary goal of CAR design is producing extracellular domains capable of targeting unique tumor cell antigens while sparing healthy tissues (150, 152). Owing to the deficit of tumor-specific antigens, lineage-specific antigens have been a key focus in CAR T cell development. Under investigation are promising candidates like CD19 (153, 154), GD2 (130, 155), GPC-3 (128), CD123 (131, 156), CD5, CEA, CD20 (10), B7H3 B7H3 (157), and PSCA (151) (**Table 2**). While CD19-targeting CAR-T products have earned FDA approval for treating B-cell lymphoma and leukemia, they carry risks, like CRS, neurotoxicity, and B-cell aplasia, primarily due to on-target off-tumor toxicities (152, 153). Interestingly, γδ anti-CD19 CAR-T cells have been reported to produce fewer inflammatory cytokines compared to their αβ counterparts, suggesting a potential decrease in cytokine-mediated side effects (90).

**Figure 3:**
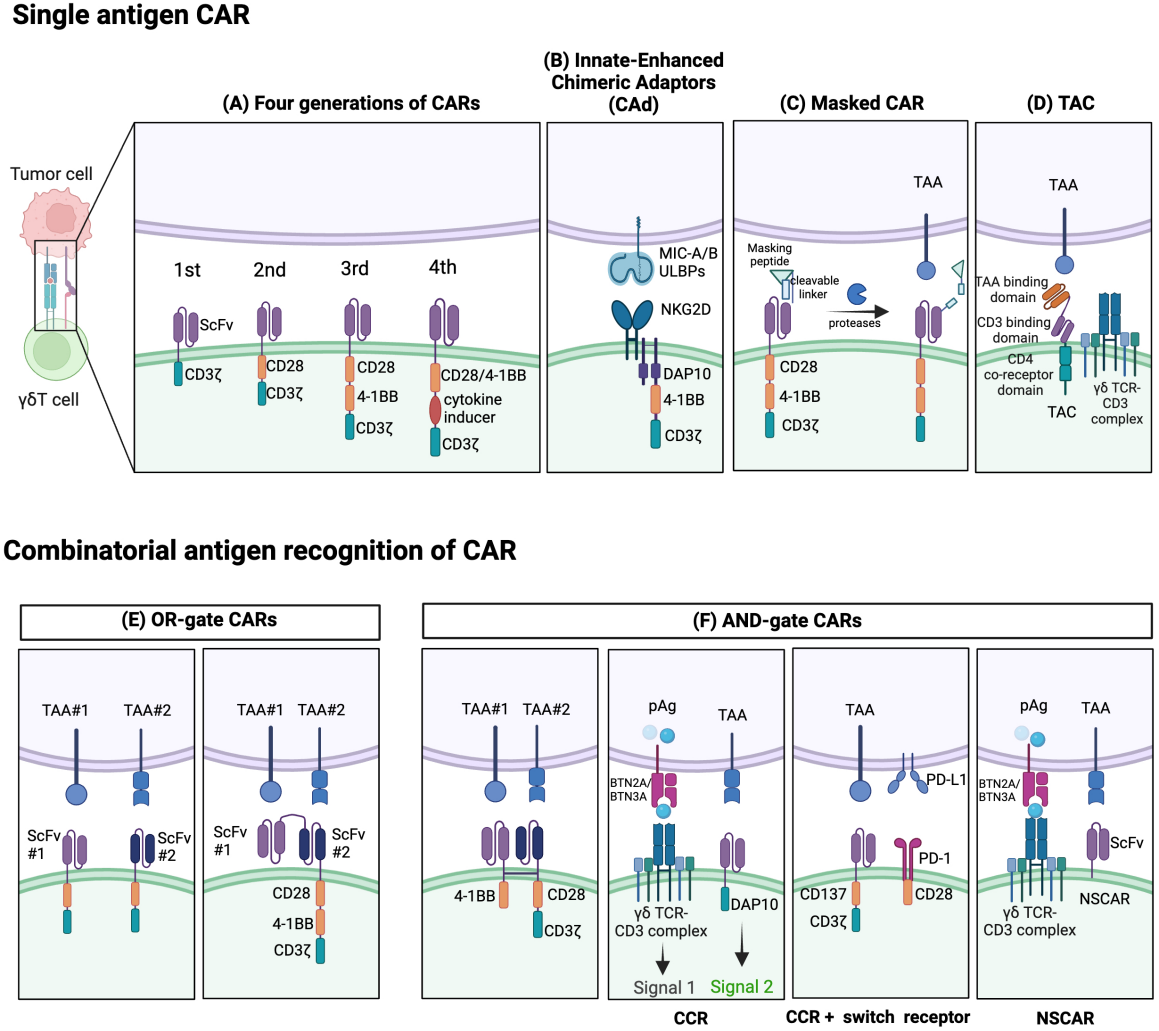
Established strategies for CAR- γδT cells. Single-antigen CAR recognition: **(A)** Conventional CARs are classified as first-, second-, third-, or fourth generation depending on their number of costimulatory domains. **(B)** Innate enhanced DAP10 chimeric adaptor (CAd), combined with 4–1BB and modified CD3ζ co-stimulation, enhances tumor targeting through endogenous NKG2D receptors. **(C)** The masked CAR (mCAR) incorporates a masking peptide. When proteases are present in the tumor microenvironment (TME), the linker is cleaved, releasing the masking peptide, and activating the CAR. This mechanism helps reduce on-target off-tumor toxicity. **(D)** A T cell antigen coupler (TAC) is also designed to reduce toxicity and promote more efficient anti-tumor response. It is comprised of a tumor-associated antigen (TAA) binding domain, CD3 binding domain, and CD4 co-receptor domain. Combinatorial antigen CAR recognition: **(E)** OR-gate CARs enable dual-targeting of antigens with separate single-chain variable fragment (scFv) domains. To prevent antigen escape, they can be designed to have two consecutive scFv domains connected to the standard CAR chassis. **(F)** AND-gate CARs are only activated when both antigens are present simultaneously, employing two separate receptors comprising the CD3ζ and costimulatory domains. A chimeric costimulatory receptors (CCR)-based AND-gate has its CD3ζ signaling domain from a γδTCR and can target multiple antigens which can enhance cytotoxicity and prevent tonic CD3ζ signaling. CCR can also be paired with a switch receptor which can be an inhibitor receptor such as programmed death-1 (PD-1) along with a costimulatory domain like CD28. Non-signaling CARs (NSCARs) do not possess signaling domains and utilize an antigen-specific tumor targeting mechanism. Created with BioRender.com.

However, the optimization of CAR for highly specific antigen recognition remains vital. Recent studies have investigated the incorporation of ligands like NKG2DL and inhibitory receptor programmed cell death ligand 1 (PD-L1) into CAR constructs to improve safety or efficacy (158). Some attempts have even added T cell antigen coupling (TAC) components to γδT cells, thereby redirecting them to target tumors with reduced off-tumor toxicity compared to conventional CAR-T cells (159, 160) (**Figure 3D**). Adicet Bio is working on CAR designs that target tumor intracellular antigens using their TCR-Like monoclonal antibodies (TCRLs) technology (91).

Clinical trials are in progress for CAR-γδT cells targeting various antigens such as CD19 (NCT02656147, NCT05554939), CD20, NKG2DL, CD7, CD33, CD28, and CD123 (**Table 3**). While many of these trials have yet to disclose their results, some promising preliminary findings have been reported. For instance, Adicet Bio, Inc. is testing an allogeneic CD20 CAR+ Vδ1 γδT cell called ADI-001, designed for patients with refractory B cell malignancies (NCT04735471). Their early report shows a 71% overall response rate and 63% complete response rate among patients with aggressive B-Cell non-Hodgkin lymphoma, all without the presentation of GvHD (91).

One challenge with CAR-T therapy is its potential ineffectiveness in tumors exhibiting heterogeneity or low antigen expression. Dual-specific CARs, which target two antigens concurrently, are proposed as a potential solution, although this requires further investigation (161). Other research focuses on fine-tuning other CAR components, including the intracellular signaling and transmembrane domain, with construction of Boolean logic gates for combinatorial antigen sensing. Balancing the DNA length of dual-CAR plasmids and transduction efficiency necessitates further study.

Recent innovative extracellular designs aimed at enhancing safety also include the development of ON/OFF switches like the masked CAR. Here, the antigen-binding site of CAR is coupled with a masking peptide through a protease-sensitive linker. Activation of masked CAR-T cells occurs when tumor microenvironment proteases cleave this linker, causing the masking peptide to detach, and revealing the antigen-binding site (162) (**Figure 3C**). In essence, this provides a level of control, reducing risks associated with unregulated CAR-T activation (162).

To sum up, while there are promising advancements in the development of γδT cell-based CAR-T therapies, it is critical to continue fine-tuning these interventions for increasing specificity and safety. A combination of innovative design strategies and rigorous clinical trials may bring forth the next generation of cancer immunotherapies. The hope is for these novel treatments to cure more patients, more reliably, with fewer side effects, revolutionizing the approach to cancer treatment.

#### 4.1.1 Co-stimulatory domain design and combinatorial strategies: emphasize the unique characteristics of γδT cells

Over the years, CARs have progressed through several generations, differentiated by the quantity and nature of their co-stimulatory domains, like CD28 and 4-1BB, which play a pivotal role in γδT cell activation and cytotoxic function (163) (**Figure 3A**). Initial designs of CAR γδT cells were largely based on pre-existing CAR-αβT designs, failing to capitalize on the unique benefits of γδT cells due to a dearth of knowledge on the fundamental CAR signaling mechanisms in γδT cells. CAR-αβT cells recognize tumor cells through the CAR pathway while completely bypassing the αβTCR. Meanwhile, in CAR-γδT cells, the inherent γδTCR signal can synergize with logic-gated CARs, providing MHC-independent cytotoxicity and downstream CD3ζ signals. Besides, CAR-γδT cells retain multiple activating NK receptors alongside CAR and TCRγδ, potentially enhancing recognition and activation. In the tumor immunoescape setting, CAR-γδT cells have been proved the ability to recognize antigen-negative tumor cells in CAR-independent manner (125). CARs designed for γδT cells can also incorporate γδT cell-specific signaling domains, such as NKG2D-DAP10, as an intracellular costimulatory domain for activation. Despite this development, contemporary research on CAR γδT cells predominantly employs second or third-generation designs. It has been observed, though, that single antigen recognition in these CARs leads to poor discrimination between tumor and healthy cells, contributing to on-target off-tumor toxicity. Furthermore, CAR-T cells exhibit strong limitations in treating T cell malignancies due to difficulties like lethal T cell aplasia and CAR-T cell fratricide stemming from shared target antigens (129). Even extending CAR T cell therapies to T cell acute lymphoblastic leukemia (T-ALL) has proven challenging, despite shared molecular commonalities with B cell acute lymphoblastic leukemia (B-ALL).

Moreover, CARs providing both CD3ζ stimulus and CD28 co-stimulation are prone to tonic signaling, leading to functional exhaustion and impaired CAR-T cell function. A unique construct called ADI-925 has been developed by Adicet Bio to help tackle this. It incorporates an enhanced intracellular DAP10 chimeric adaptor (CAd), 4–1BB, and a modified CD3ζ co-stimulation, designed to enhance tumor targeting through endogenous NKG2D receptors (121, 164) (**Figure 3B**).

Novel strategies are also emerging, employing Boolean logic gates (like AND, OR, AND NOT) enabling CAR-T cells to detect multiple antigens, reducing off-tumor toxicity and minimizing potential antigen escape (**Figures 3E, F**). Dual-targeting CAR γδT cells, like those targeting GD2 and PTK7 in preclinical studies for neuroblastoma, were developed to help avoid antigen escape through an OR-gate strategy) (165). Though promising, tandem bispecific OR-gate CAR-T cells may induce excessive CD3ζ signaling during co-stimulation, necessitating alternative strategies (165).

Bi-specific CARs with split co-stimulatory signals and a shared CD3ζ domain have emerged as another strategy, allowing for optimal CAR-T cell activation only when both antigens are simultaneously present (161, 166). Furthermore, ideas like chimeric costimulatory receptors (CCRs), also known as recognition-based logic-gated CAR, and non-signaling CARs (NSCARs) have been proposed to mitigate on-target off-tumor toxicity (123, 129). CCRs, traditional CARs without CD3ζ signaling domain, provide co-stimulation whilst avoiding tonic CD3ζ signaling of γδT cells. Thus, these reduce on-target off-tumor toxicity by separating co-stimulatory input from the primary TCR signal (129). Moreover, CCRs have the potential to target malignant cells while sparing healthy tissues in scenarios where the target antigen is broadly expressed (123, 129). Fisher et al. developed a co-stimulation-only CAR, wherein the CAR is fit only to provide co-stimulation, thereby restricting tonic signaling but still facilitating rapid downstream response upon activation (164). Concurrently, CAR-γδT cytotoxicity can be selectively triggered by both the CAR signal and the inherent γδTCR signal when encountering cancer cells (130). CCR can also function as a switch chimeric receptor combined with a second-generation CAR (**Figure 3B**). The switch receptor typically includes an inhibitory receptor (e.g. PD-1 or TIGIT) and an intracellular costimulatory signal (167). For instance, the PD-1-CD28 construct as anti-PD-L1 CCR can potentially convert the inhibitory signal into an activating one (167). Such a design can accelerate activation of CAR-T cells and improve their survival in the immunosuppressive tumor microenvironment (167, 168). On the other hand, NSCARs capitalize on γδT cells’ MHC-independent cytotoxic capacity while eliminating all CAR signaling domains (129). This results in antigen-specific tumor cell-targeting capability without influencing T cell activation, as demonstrated by Fleischer et al. with CD5-NSCAR- and CD19-NSCAR-engineered γδT cells, designed specifically for T-ALL and B-ALL relief (129).

Despite the promise of these technologies, factors like NSCAR shedding on γδT cells and antigen downregulation in target cells have somewhat limited their translational application in clinical therapies. Additionally, the necessity of intracellular signaling domains in CAR design is being reconsidered when applied to γδT cells. Deletion of these domains can potentially allow for the transduction of multiple NSCARs, due to a decrease in overall CAR size.

In conclusion, recent years have seen significant expansion in the approaches to T cell engineering, including innovations such as synNotch receptors, iCAR, and several others (158, 169). However, the design and development of CARs for γδT cells haven’t kept pace. A deeper understanding of γδT cell cytotoxicity mechanisms and further research into these novel CAR structures will be critical in achieving maximum safety and efficacy, thereby unlocking the full potential of CAR γδT cell therapies.

#### 4.1.2 CAR transduction methods

The primary methodologies for CAR-T therapy involve permanent DNA-based transfection methods that include viral transduction (using lentiviruses or retroviruses) and non-viral transfection, typically utilizing transposon systems like Sleeping Beauty and Piggy Bac (170) (**Table 2**). While lentiviruses and retroviruses are commonly used, concerns about their safety, predominantly due to their immunogenic properties, and their complex and costly manufacturing processes may limit their utility. Despite these concerns, retrovirally-modified CAR-T cells have proven tolerable safety profiles in extensive clinical trials (171). However, the transduction of γδT cells has been challenged due to their relatively limited proliferation and susceptibility to AICD compared to that of αβT cells (172). Gammaretroviruses necessitate active cell proliferation for the penetration of viral nucleic acids into the nucleus. This poses a challenge for the transduction of γδT cells compared to αβT cells, demanding necessary specific proliferative stimuli for effective γδT cell transduction (172).

Simultaneously, advancements are being made in non-viral technologies to address some drawbacks associated with viral transductions, such as potential oncogenesis, immunogenicity, and high cost (170). Non-viral transposon vectors possess simpler manufacturing processes, cost efficiency, enhanced safety, stable integration of large sequence (>10 kb), but often face efficiency challenges (173). These non-viral integrative vectors rely on temporary cell pore formation or endocytosis, accomplished via various chemical or physical techniques, including electroporation and liposomes (174).

More recently, non-permanent gene transfer methods that utilize non-integrating gene delivery like mRNA-based CAR expression have started to gain traction (154). The utilization of mRNA in CAR-T cells allows for a “biodegradable” approach, in which the cell’s potency is short-term. The use of mRNA electroporation was first applied in early stages of αβ CAR-T development, but initial clinical trials indicated a lack of efficacy, potentially due to the poor quality and quantity of patient-derived autologous αβT cells (NCT02623582). This led researchers to explore the use of allogeneic Vγ9Vδ2 T cells from healthy donors. Investigations revealed that after mRNA electroporation, CAR expression persisted for up to 120 hours, with peak expression at the 24-hour mark (175). Enhanced anti-AML activity of mRNA-based anti-CD123 γδ CAR-T was observed both *in vivo* and *in vitro* (131). Despite these promising results, the transient nature of receptor expression means that further applications may need to employ strategies such as repeated or intratumoral injections to ensure therapeutic efficacy. Future advancements in CAR γδT cell therapy may favor non-viral integrating and lipid nanoparticles technological platforms (170).

In the domain of hematological malignancies, CAR γδT cell therapy holds formidable promise. However, the development of universal CAR γδT cells capable of effectively treating solid tumors remains a pressing need, necessitating ongoing research to overcome the physical and immunological challenges associated with solid tumor immunity. Given the unique stimulatory signals and recognition mechanisms of γδT cells, it is evident that the design of CARs for these cells needs to undergo revisions and refinements as our understanding of their biological mechanisms deepens. In essence, while there has been substantial progress in the field of CAR γδT cell therapy, future work that ensures the safety, efficacy, and broad applicability of this promising therapy modality, especially in the context of solid tumors, remains a critical need in the field.

### 4.2 Cell engagers or bispecific antibodies: easier ways to enhance γδT cells recognition

**Figure 4:**
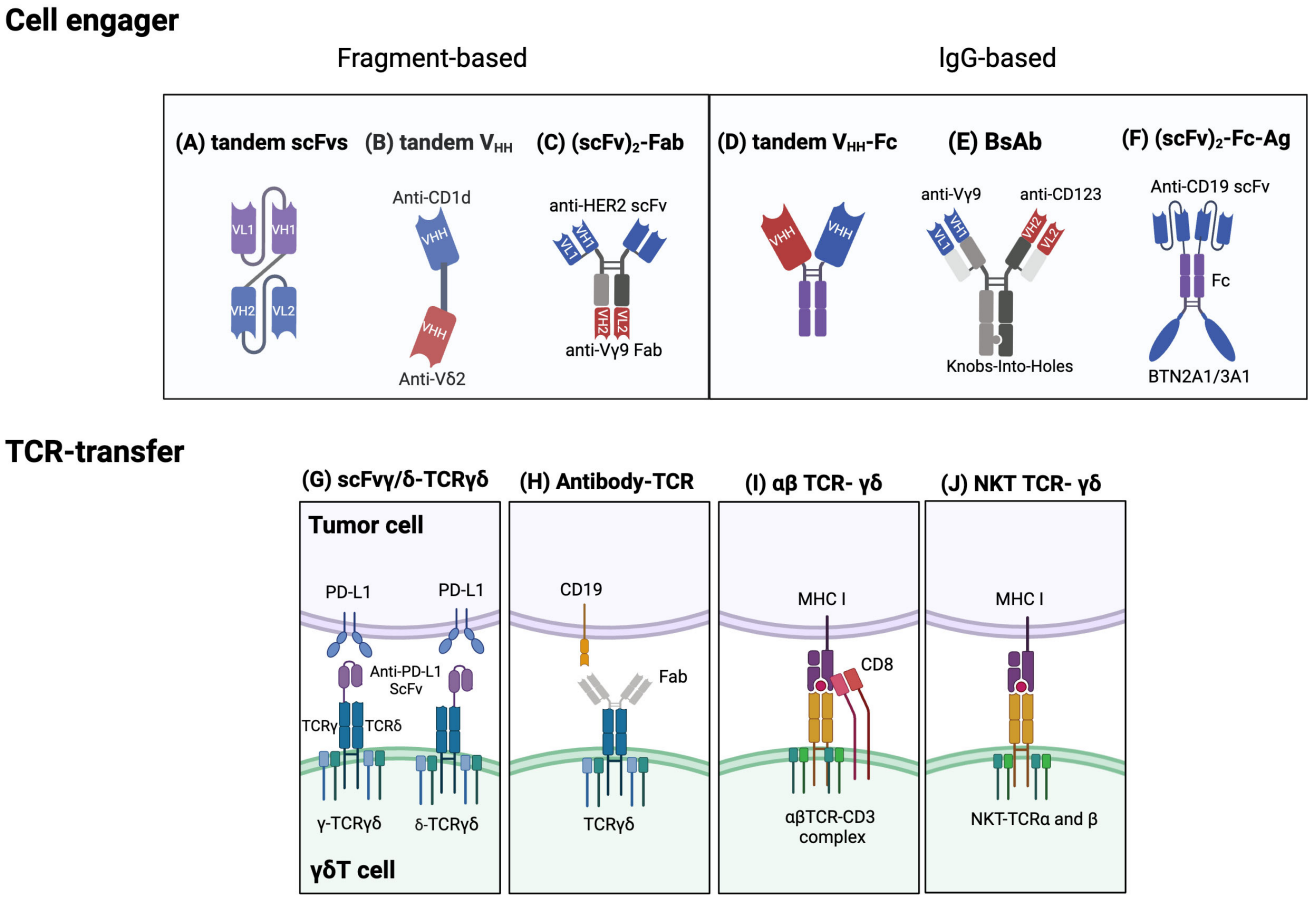
Established strategies for engineering γδT cells. Cell engager designs: Fragment based cell engagers include tandem single-chain variable fragment (scFv), tandem variable heavy chain (VHH), and (scFv)2-Fab. **(A)** A tandem scFv antibody comprises two different scFvs joined by a linker. **(B)** Tandem VHH is depicted as a bispecific T cell engager (bsTCE) with an anti-CD1d VHH linked to an anti-Vδ2 VHH. **(C)** An example of (scFv)2-Fab antibody, Her2/Vγ9, is composed of an anti-Vγ9 Fab domain and two anti-Her2 scFvs. This design selectively recruits γδ T cells and enhances cytotoxicity. IgG based cell engagers encompass tandem VHH-Fc, bispecific antibodies (BsAb), and (scFv)2-Fc-Ag. **(D)** Tandem VHH-Fc antibodies involve two VHHs linked to a Fc domain. **(E)** One type of BsAb connects an anti-Vγ9 domain and an anti-CD123 domain via Knobs-into-holes heterodimerization technology. **(F)** (scFv)2-Fc-Ag is shown as an anti-CD19 scFv connected to a BTN2A1/3A1 domain via an Fc linker. Engineering γδTCRs and transferring specific αβT-TCR or NKT-TCRs into γδT cells: **(G)** One approach to engineering γδTCRs is to fuse an anti- programmed cell death ligand 1 (PD-L1) scFv to either the γ or δ chain of γδTCR to limit T cell exhaustion. **(H)** Another approach is an antibody-TCR, such as an anti-CD19 Fab domain linked to a γδTCR. **(I)** αβTCRs and CD8 αβ genes can be transferred to γδT cells to enable targeting specific tumor cells and avoid TCR mispairing. **(J)** Natural killer T (NKT) cell-derived αβTCRs can also be transferred into γδT cells to enhance proliferation, IFN-γ production, and antitumor effects. Created with BioRender.com.

Cell engagers and bispecific antibodies have become an increasingly attractive immunotherapeutic method for enhancing the anti-cancer activity of γδT cells. Bispecific T cell engagers (bsTCEs) are specially designed antibodies, each having two separate binding areas aimed at individual components like tumor-associated antigens (TAAs) and the TCR complex (Vδ2 or Vγ9) (176). The flexibility of bsTCEs allows for varied applications, such as MHC-independent targeting of TAAs by γδT cells, immune checkpoint modulation, and controlling inflammatory and other signaling pathways (176). These functionalities provide several unique advantages, including their small molecular size and high versatility, eliminating the need for additional co-stimulatory signals for T cell activation, low picomolar range for the half-maximal effective concentration (EC50), effectiveness against both blood-borne and solid tumors, excellent safety profile, and efficient and cost-effective production (177). Most frequently, cell engagers incorporate a fragment-based design or lgG/lgG-like formats (136, 137). Fragment-based designs principally modify constructs such as scFv (178), Fab (135), or single-domain antibodies (sdAbs, also known as V_HH_) (176) into their binding regions (**Figures 4A-C**). sdAbs, originating from the variable domain of heavy-chain-only antibodies, have attracted attention because of their unique features, including small size, target specificity, and minor immunogenicity (179). Currently, cell engagers can be applied both as stand-alone therapies and in partnership with allogeneic γδT cells to generate readily available products.

The first CD3-targeting bsTCEs, exemplified by blinatumomab and Tebentafusp, yielded significant positive outcomes in B-cell malignancy and melanoma patients during clinical trials (NCT03070392) (180). However, adverse effects like CRS and immune effector cell-associated neurotoxicity syndrome (ICANS) constrained their clinical usage (177). Further, CD3-targeting bsTCEs may unintentionally activate other CD3+ T cell subsets, which could depress tumor-specific immune responses (137). As a category of innate T cells, γδT cells present a logical choice for engagement to reduce CRS and off-tumor toxicity.

The successful usage of bsTCEs in LAVA Therapeutics’ Gammabody platform, employing tandem single-domain antibodies (V_HH_s) (**Figure 4D**), exemplifies their potential. These include EGFR-Vδ2, CD1d-Vδ2, CD40-Vδ2, and PSMA-Vδ2 bsTCEs (**Table 3**). EGFR-Vδ2 bsTCEs have displayed compelling activation of Vγ9Vδ2 T cells which induce cytotoxicity against EGFR+ tumor cells (137, 154). The CD1d-Vδ2 bsTCE, or LAVA-051, has shown anti-tumor potential against hematological malignancies expressing CD1d in preclinical models (176). Its specificity for NKT and Vγ9Vδ2-T cells, alongside low-nanomolar range EC50 values *in vitro*, further demonstrates its potential (176).

Bispecific antibodies (bsAbs) comprise a class of engineered antibodies with two distinct binding sites, setting them apart from traditional antibodies (181) (**Figures 4D-F**). These antibodies, as exemplified by anti-Vγ9/CD123 bsAbs, selectively rally Vγ9+ γδT cells, promoting cell conjugate formation between γδT cells and AML cells (136). As such, these cell engagers can enhance Vγ9Vδ2+ T cell cytotoxicity against B-cell lymphoma, particularly when accompanied by a co-stimulatory signal pair (178). ImCheck Therapeutics’ humanized anti-BTN3A antibody, ICT01, serves as another example. It operates by recognizing three distinct BTN3A forms and prompting their activated conformation, thereby selectively activating Vγ9Vδ2 T cells in an antigen-independent manner (182).

In a phase I/II clinical trial, ICT01 showed tolerable safety profile and increased infiltration of Vγ9Vδ2 T cells into tumor tissue in patients with advanced solid tumors (NCT04243499). (**Table 3**) Besides, LAVA-1207 (PSMA-Vδ2 bsTCEs) has shown a favorable safety profile and clinical symptom improvement (decreased PSA level) in a Phase 1/2a clinical trial involving metastatic castration-resistant prostate cancer (mCRPC) patients (N=20, NCT05369000) (137).

Notably, as cell engagers depend on the activation and migration of the patient’s inherent γδT cell pool, initial Vγ9δ2T cell counts could be a useful predictor for clinical outcomes. Take, for instance, a melanoma patient with a high baseline count of circulating Vγ9Vδ2 T cells who showed considerable tumor infiltration of Vγ9+ T cells post ICT01 administration (182). Cell engagers can also be combined with γδT cell-based therapies to develop easily available TAA-targeting γδT cell products (148).

Acepodia’s technology, for instance, conjugates antibodies to cells to create products like ACE1831, which is the CD20-targeting γδT cells (148). This product is currently under phase I trial for patients with relapsed/refractory B-cell lymphomas (NCT05653271). Other products, ACE2016 (EGFR-targeting γδT) and ACE1708 (PD-L1-targeting γδT), are in the preclinical exploratory stage (183).

In conclusion, while cell engagers and bispecific antibodies present significant potential compared to CAR-T therapy, their definitive superiority is yet to be determined. Like CAR-T therapy, cell engagers also encounter hurdles such as immune escape owing to loss of target antigen expression and an immunosuppressive tumor microenvironment. Further research is needed to modify cell engagers specifically for γδT cells, paving the way for effective treatments in the future.

### 4.3 TCRs engineering or transfer: a highly specific and reproducible manner

Harnessing natural receptors through the engineering or transfer of T cell receptors (TCRs) serves as an alternative approach to the use of synthetic ones. The transduction of cancer-specific TCRs is an appealing strategy for generating large volumes of readily available, antigen-specific T cells. Transferring cancer-specific αβTCR engenders T cell specificity, simplifying procedures compared to isolating specific T cell subsets. However, the transgenic transfer of αβTCRs to other αβT cells runs the risk of triggering TCR competition and mispairing. Recognizing these limitations, γδT cells are appreciated as safe and ideal carriers for antigen-specific effector cells because TCR-α and -β chains can’t pair with TCR-γ and -δ chains (138, 184). To produce cytotoxic γδT cells capable of attacking tumor cells and secreting cytokines via αβ and γδTCR-dependent activity, one can isolate tumor antigen-specific αβ CD8+ cytotoxic T lymphocytes and clone their TCR αβ genes (138) (**Figure 4I**). However, a notable reduction in γδTCR expression post αβTCR transduction was observed, likely due to competition for limited CD3 molecules (138).

Van der Veken et al. demonstrated that αβTCR -transduced γδT cells display sustained *in vivo* endurance and can elicit a recall response (139, 184). More so, infusing αβTCRs from invariant natural killer T (iNKT) cells into γδT cells can create bi-potential T cells with NKT cell functionality (143) (**Figure 4J**). Other research endeavors are concentrated on transferring γδTCR to αβT cells to leverage the superior understanding of their effects and memory function mechanisms (185). One product, GDT002, which contains Vγ9Vδ2TCR-expressing αβT cells, allows αβT cells to detect augmented phosphoantigens in stressed or malignant cells (185). An ongoing phase 1/2 study is investigating GDT002’s safety and tolerability in patients with multiple myeloma. Furthermore, strategies for engineering TCRγδ involve fusing with single-chain variable fragments (scFv) or Fab fragments from antibodies. For example, one study used CRISPR/Cas9 to fuse an anti-PD-L1 scFv to the TCRγ or δ chain in activated γδT cells, creating scFv-γ/δ-TCRγδ cells that showcased anti-tumor capacity akin to traditional CAR-T cells (186) (**Figure 4G**). Alternatively, the Fab domain of an antibody can be connected to the C-terminal signaling domain of the γ and δ chains of the TCR, creating an antibody-TCR construct (187) (**Figure 4H**). The use of the TCR alongside endogenous costimulatory molecules can lower co-stimulation input compared to CAR constructs, thus diminishing cytokine release and mitigating the exhaustion phenotype (187). Anti-CD19 Fab – TCR-γδT cells or ET019003, for instance, have displayed similar anti-tumor actions against B-cell lymphoma as CAR-T cells *in vivo* (187).

Promisingly, a phase I clinical trial (NCT04014894) indicates that, aside from showing agreeable safety profiles, ET019003 has achieved an impressive clinical response rate (87.5%) among patients with relapsed or refractory diffuse large B-cell lymphoma (188). However, TCR gene transduction or engineering research has somewhat stagnated in recent years, possibly due to complex manufacturing processes involved. In summary, the exploration of novel therapeutic approaches incorporating γδT cells continues to expand, with significant potential for future cancer treatment innovations.

### 4.4 Combination therapy

CAR-T cell therapy has proven extremely promising for treating hematologic malignancies. However, distinct issues related to the immunosuppressive microenvironment of solid tumors require further refinement and personalization of this approach. A potential solution could be combination therapies that adequately address the complexity of solid malignancies.

The concurrent usage of CAR-T/bsTCE therapies and immune checkpoint inhibitors is recognized as a potentially effective strategy to overcome immune system suppression. Exhaustion status, marked by the upregulation of inhibitory receptors, can potentially compromise the therapeutic efficacy of CAR-T cells (189). In a murine model of bone metastatic prostate cancer, γδ CAR-T cells persisted in the tumor-bearing tibia for approximately 21 days post-infusion. However, these cells exhibited an upregulation of PD-1 expression while simultaneously losing expression of activation markers (151). Consequently, the combination of therapies such as ICT01 and pembrolizumab, an anti-PD1 antibody, exhibited favorable safety profiles in a phase I clinical trial (NCT04243499). This suggests that the co-administration of CAR-T/bsTCE therapy with anti-PD-1/PD-L1 antibodies could potentially boost treatment benefits (151).

Chemotherapy and radiotherapy, owing to their immune-sensitizing attributes, are plausible options for combination therapy with immunotherapy (190). Temozolomide (TMZ), a chemotherapy mainstay for glioblastoma (GBM), transiently heightens the expression of stress-associated antigens such as NKG2DL on tumor cells. Engineering γδT cells to express the methylguanine DNA methyltransferase (MGMT) can thus potentially confer TMZ resistance, enabling the engineered cells to operate efficiently despite the presence of therapeutic concentrations of chemotherapy. The amalgamation of TMZ and MGMT-modified autologous γδT cells, or drug resistant immunotherapy (DRI), showed improved survival outcomes in a model of high-grade gliomas compared to monotherapy (124).

In a phase I clinical trial, INB-200 (an example of DRI) displayed a favorable safety profile, extended progression-free survival (PFS), and presented no dose-limiting toxicities, CRS, or neurotoxicity in glioblastoma multiforme patients (NCT04165941). As a result, autologous DRI- γδT cells (INB-400) have proceeded to a phase II clinical trial, and MGMT-modified allogeneic γδT cells (INB-410) are currently undergoing a phase Ib clinical trial (NCT05664243). The product INB-400/410, developed by IN8bio, has been granted FDA Orphan Drug Designation for the Treatment of Newly Diagnosed Glioblastoma.

As it stands, most approved combination immunotherapies largely rely on a combination of immune checkpoint inhibitors (ICIs) and have emerged as first-line treatments for several major cancer types (191). The future of combination immunotherapies with γδT cells likely extends beyond ICI-based approaches, aiming for control and eradication of established tumors. Further research in this area will be instrumental in harnessing the full therapeutic potential of γδT cells.

## 5 Challenges and limitations

Tapping into the potential of genetically engineered γδT cells holds the promises of breakthroughs in cancer immunotherapy, albeit with scientific and technical hurdles. The multifaceted nature of γδT cell biology coupled with the complexities of genetic manipulation throws inevitable challenges in the way of optimizing therapeutic potential.

The extensive heterogeneity of γδT cells, which includes various subsets with distinct antigen recognition patterns, homing properties, and effector functionalities, presents a significant challenge in standardizing genetic engineering strategies (192). Additionally, our understanding of the γδTCR repertoire lags behind that of αβTCRs (141). Although cell engagers and bispecific antibodies have shown potential to robustly activate γδT cells, effective signal optimization is still underway (178). Certain constraints of gene-engineering, such as the need for CD8 or other co-stimulators which γδT cells lack, and the intricate manufacturing processes involved, serve as significant obstacles (138). Although gene-transduction techniques, such as mRNA electroporation and lentiviral transduction, have seen noticeable advancements over the past years, the efficiency of integrating genes into γδT cells using either viral or non-viral vectors is yet to reach optimal levels. mRNA electroporation allows for rapid expression and poses fewer risks of insertional mutations, while also being associated with lower cellular toxicity. However, this method only provides transient expression of CARs, requiring multiple infusions of CAR T-cells and an extension of their cytotoxic lifespans from a therapeutic perspective (154). On the other hand, lentiviral transduction, often considered time-consuming, also carries the risk of damaging essential genes or regulatory sequences during the period required for expression (193–195)..

In comparison to αβ CAR-T cells, γδ CAR-T cells often present less complete clearance of tumor cells *in vivo*. This characteristic could be attributed to reduced persistence of γδ CAR-T in the immunosuppressive microenvironment (125, 196), necessitating multiple infusions and a large supply of γδ CAR-T cells. Furthermore, CAR-T cells could potentially contribute to antigen loss in target cells, resulting in diminished antigen density (197).

While the introduction of bispecific T cell engagers has propelled cancer immunotherapy, especially against hematological malignancies by offering an easy and cost-effective treatment option, their efficacy remains undermined by co-triggering of immunosuppressive T cell populations, such as regulatory T cells (Tregs) (137). Even though the combination of CAR and bispecific γδT cell engagers has shown promising results towards improving anti-tumor efficacy and reducing cytotoxicity, the tumor cells’ ability to evade the immune system strengthened by γδT cells is still under investigation (198).

Interestingly, γδT cells, under certain conditions, may also promote tumor growth (199, 200). This trait might be influenced by the TME or interactions with other immune cells. γδT cells have been known to promote tumor growth by producing IL-17, a process influenced by factors such as TME-related metabolism, microbial products, and inflammatory cells (201, 202). Considering the association of γδT cells with autoimmune diseases, a thorough investigation of their long-term clinical outcomes is essential when their activation or suppression is incorporated into treatments (203).

Implementing engineered γδT cell immunotherapy in a clinical setting presents its own set of challenges. Identifying suitable patients and healthy donors and creating standardized monitoring guidelines are crucial. Determining the correct dosage—whether based on body weight or the number of cells per infusion—and understanding its relation to treatment success is a significant hurdle. There is also a pressing need to address the risk of disease recurrence post-treatment, bolster the therapy’s durability, and decide whether to opt for monotherapy (with a single or several doses) or a combination approach (204). In addition, as production is resource-intensive and coupled with strict regulatory, ethical, and safety considerations, and high costs. Thus, widespread access to this form of therapy is limited. To fully employ the potential of γδT cell therapies, extensive research and collaboration are necessary.

## 6 Conclusions and future perspectives

γδT cell-based immunotherapies represent a promising frontier in cancer treatment, introducing innovative approaches to overcome the limitations of traditional therapies. The development of gene-engineering strategies, such as CAR T therapy, bispecific antibodies and cell engagers, and TCR gene transfer, has significantly advanced the efficacy of γδT cells, addressing their challenges in abundance, expansion, and targeting efficiency. Despite these strides, hurdles such as the nuanced understanding of γδT cell behaviors, targeting solid tumors effectively, and preventing post-treatment relapse persist.

The remarkable potential of γδT cell therapies lies in their ability to offer a paradigm shift in cancer treatment, utilizing their unique properties for more precise, potent, and personalized interventions. Their versatility in recognizing cancer cells without MHC restriction provides a substantial advantage in reducing the risk of immune escape and addressing tumor heterogeneity.

Looking ahead, research must focus on understanding γδT cells’ metabolic needs and cytokine profiles within the tumor microenvironment to enhance their antitumor activity. Additionally, it is critical to develop strategies that improve the persistence of CAR γδT cells and maintain target antigen visibility, ensuring long-term therapeutic success. The exploration of Vδ1 subsets and the creation of iPSC-derived γδT cells hold promise for developing universally applicable CAR γδT cell therapies. Furthermore, optimizing the engineering of γδT cells for safer and more efficient delivery, coupled with the strategic combination of these therapies with other treatments, will enhance efficacy and durability.

Emphasis should also be placed on designing therapies that reduce the risk of relapse and increase sustainability. Regulatory, manufacturing, and logistical challenges will need to be addressed to facilitate the clinical translation of these therapies. The ultimate goal is to harness the full therapeutic potential of γδT cells, offering new hope to patients with various types of cancer.

The future of γδT cell immunotherapy lies in the convergence of molecular biology, genetic engineering, and clinical research. As our understanding evolves, so will the potential of γδT cells as a powerful tool in the arsenal against cancer, paving the way for more effective, tailored, and sustainable cancer treatments.

## Author contributions

MY: Writing – original draft, Writing – review & editing. WW: Writing – original draft, Writing – review & editing. IH: Writing – review & editing. JH: Writing – review & editing. ZY: Writing – review & editing. AB: Writing – review & editing.
